# Supplementary material for: Trimethylamine N-Oxide Promotes Autoimmunity and a Loss of Vascular Function in Toll-like Receptor 7-Driven Lupus Mice
Source: Antioxidants (Basel). 2021 Dec 30;11(1):84. doi: 10.3390/antiox11010084 (PMC8773414; doi:10.3390/antiox11010084)
Supplement: Supplementary file 1 [file antioxidants-11-00084-s001.zip › antioxidants-1508419-supplementary.pdf]

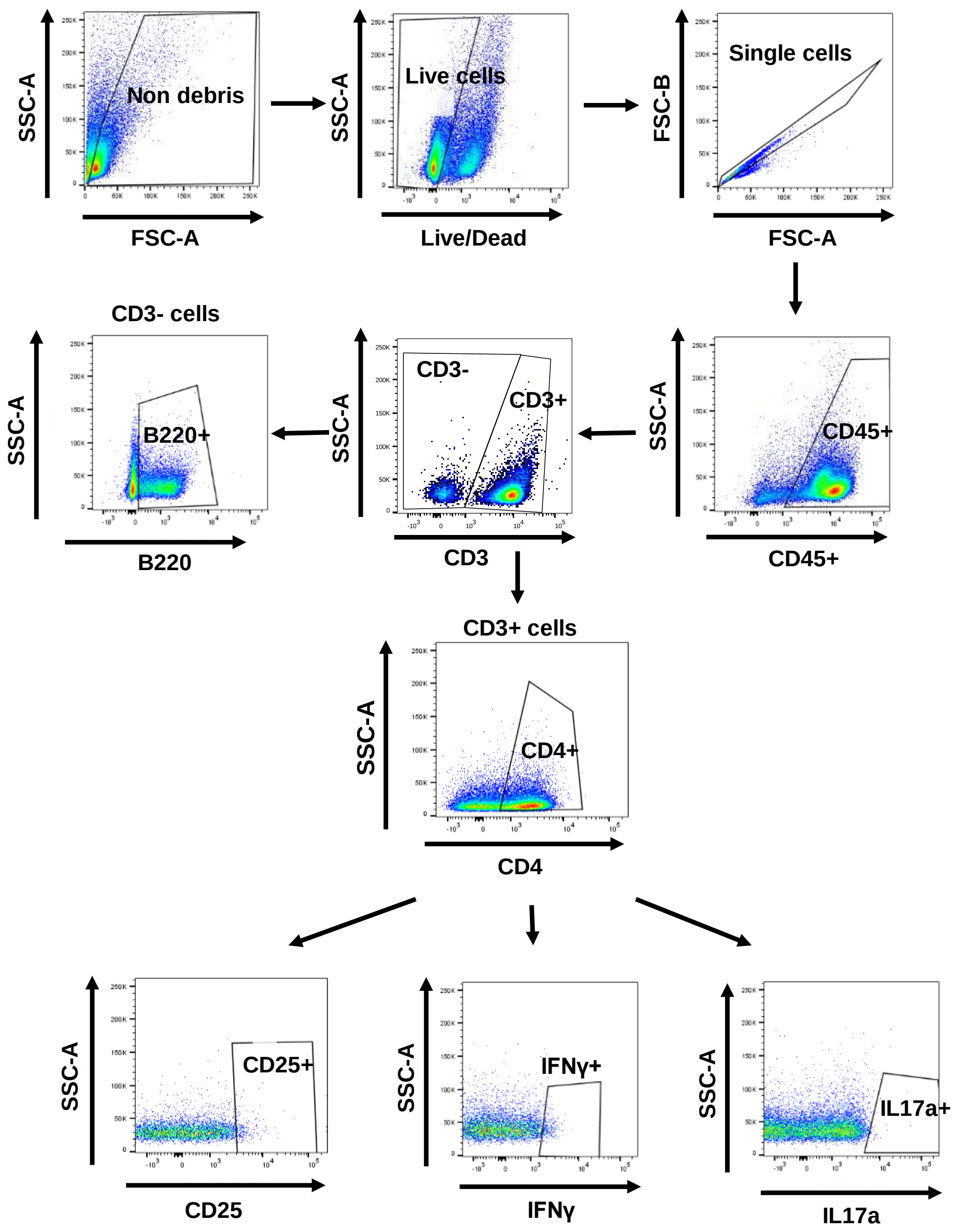

Figure S1. Gating strategy for flow cytometry.

Table S1. Oligonucleotides for real-time RT-PCR

Accession number	mRNA targets	Descriptions	Sense	Antisense
NM_008713.4	*eNOS*	endothelial nitric oxide synthase	GCATGGGCAACTTGAAGAGTG	GGGAACACTGTGATGGCTGA
NM_015760.5	*NOX4*	NOX-1 subunit of NADPH oxidase	GGATCACAGAAGGTCCCTAGC	TTGCTGCATTCAGTTCAAGG
NM_001286037.1	*P47phox*	P47phox subunit of NADPH oxidase	TCTTCAAAGTGCGGCCTGAT	TGCCACGGTCATCTCTGTTC
NM_010902.4	*NRF2*	Nuclear factor-erythroid 2-related factor 2	CTGAACTCCTGGACGGGACTA	CGGTGGGTCTCCGTAAATGG
NM_016679.4	*KEAP-1*	Kelch-like ECH-associated protein 1	CCCATGAGGCATCACCGTAG	CATAGCCTCCGAGGACGTAG
NM_008706.5	*NQO-1*	NADPH quinone dehydrogenase 1	CATTCTGAAAGGCTGGTTTGA	CTAGCTTTGATCTGGTTGTCAG
NM_010442.2	*HO-1*	Hemo-oxigenase-1	CCTCACTGGCAGGAAATCATC	CCTCGTGGAGACGCTTTACATA
NM_145827.4	*NLRP3*	NLR family pyrin domain containing 3	CCTGACCCAAACCCACCAGT	TTCTTTCGGATGAGGCTGCTTA
NM_008361.4	*IL-1β*	Interleukin-1 beta	GCTACCTGTGTCTTTCCCGT	CATCTCGGAGCCTGTAGTGC
NM_010493.3	*ICAM-1*	Intracellular adhesion molecule 1	GGTTCTCTGCTCCTCCACAT	CCTTCCAGGCTTTCTCTTTG
NM_010503.2	*IFNalpha*	Interferon alpha	GACTTTGGATTTCCCCTGGAG	AAGCCTTTGATGTGAAGAGGTTC
NM_001289726.1	*GAPDH*	Glyceraldehyde-3-phosphate dehydrogenase	TGCACCACCAACTGCTTAGC	GGATGCAGGGATGATGTTCT
